# Research landscape, hotspots, and evolutionary trends of knee osteoarthritis and oxidative stress: a multidimensional bibliometric analysis

**DOI:** 10.3389/fmed.2026.1819154

**Published:** 2026-06-10

**Authors:** Yuhang Zhang, Erkun Yang, Rongfeng Lu, Bin Xie, Dianhe Du, Yuankun Xu

**Affiliations:** 1 Orthopedics College, Guizhou University of Traditional Chinese Medicine, Guiyang, Guizhou, China; 2 Department of Orthopedics, The First Affiliated Hospital of Guizhou University of Traditional Chinese Medicine, Guiyang, Guizhou, China

**Keywords:** bibliometrics, knee osteoarthritis, oxidative stress, research landscape, visual analysis

## Abstract

**Objective:**

This study aims to analyze the research status, hotspots, and development trends in the field of KOA and oxidative stress using a multidimensional bibliometric approach.

**Methods:**

Literature related to KOA and oxidative stress was retrieved from the Web of Science Core Collection. CiteSpace, VOSviewer, and RStudio were employed to analyze publication volume, scientific collaboration networks, keyword co-occurrence, clustering, and the characteristics of hotspot shifts, with the goal of revealing the overall research landscape of KOA and oxidative stress.

**Results:**

Research volume on KOA and oxidative stress has entered a phase of rapid expansion, peaking in 2024. While China leads in total publication output, its relatively low centrality (0.06) combined with a low global institutional network density (0.0061) reveals a “high-output, low-integration” pattern, indicating significant potential for enhanced international synergy. Key knowledge hubs are centered around institutions like Nanjing University of Chinese Medicine and Universidade da Coruña, with leading scholars such as Blanco FJ and Li J driving the field’s intellectual core. Keyword clustering identifies a three-dimensional thematic landscape: nutritional intervention (e.g., vitamin E), mechanistic exploration (e.g., cell senescence), and therapeutic investigation (e.g., ozone). The research evolution follows a clear trajectory of “foundational validation → mechanistic refinement → translational expansion,” with current frontiers shifting from localized joint mechanisms toward systemic-local interactions.

**Conclusion:**

The field of KOA and oxidative stress is characterized by an internationally collaborative yet resource-fragmented research landscape. Its evolution follows a logic of initial foundational validation, followed by mechanistic refinement, and finally translational expansion. Future efforts should strengthen interdisciplinary collaboration and promote multicenter clinical translational research, which holds promise for developing more targeted and effective novel strategies for the diagnosis and treatment of KOA.

## 1 Introduction

Knee osteoarthritis (KOA) is a chronic, progressive, and multifactorial degenerative joint disease, currently conceptualized as a pathological state involving the entire joint apparatus rather than being limited to articular cartilage degradation (1). Knee osteoarthritis is a degenerative, whole-joint disease characterized pathologically by the progressive degradation and loss of articular cartilage, subchondral bone remodeling (manifested as osteophyte formation and subchondral sclerosis), concomitant chronic synovitis, and the degeneration of periarticular soft tissues (2). The etiology of knee osteoarthritis is multifactorial, characterized by the complex interaction of systemic susceptibility factors—namely advanced age, female sex, obesity, and genetic factors (2)—acting in concert with local mechanical stressors, such as joint injury (3), prolonged mechanical overload (4), and joint malalignment (5). Clinically, KOA manifests as persistent knee pain, restricted range of motion, and functional impairment, which may lead to permanent disability and significantly compromise the quality of life and social participation of middle-aged and elderly populations (6). While traditionally conceptualized as an inescapable sequela of mechanical wear and biological senescence, the understanding of KOA has shifted toward a complex molecular paradigm. Within this framework, oxidative stress (OS)—defined as an imbalance between pro-oxidant generation and antioxidant defense—has been recognized as a pivotal pathophysiological driver governing both the initiation and clinical progression of the disease (7).

Oxidative stress represents an imbalance between oxidation and antioxidation in cells under stressor conditions, characterized by excessive reactive oxygen species (ROS) and/or depleted antioxidants (8). ROS, including hydrogen peroxide (H₂O₂), hydroxyl radicals (OH^−^), superoxide anion (O₂^−^), and nitric oxide (NO) (9), contribute to the disease process by modulating cell signaling, promoting chondrocyte apoptosis, disrupting the synthesis and degradation of the extracellular matrix, and inducing synovitis. These mechanisms collectively lead to cartilage matrix degradation and programmed cell death, establishing a vicious cycle that perpetuates joint deterioration (10, 11).

Over the past three decades, substantial scientific literature has accumulated regarding KOA and oxidative stress. However, several critical questions remain: How has this research field evolved? How have its core themes and research fronts transformed across different historical periods? Have there been any landmark “paradigm shifts” or “intellectual turning points”? Addressing these questions is crucial for comprehending the complete knowledge landscape of this field and for strategically guiding future research directions.

Bibliometrics, through quantitative analysis of extensive literature data, can identify research hotspots, analyze research fronts, predict development trends, and reveal the developmental dynamics and focus areas within a discipline (12). This study aims to employ CiteSpace, VOSviewer, and RStudio to conduct a comprehensive bibliometric analysis and construct knowledge maps of global scientific literature in the field of KOA and oxidative stress research published between 1996 and 2025. Through this data-driven approach, we strive to objectively present the evolutionary trajectory of this research domain over 30 years, uncover the phased transitions of its research hotspots, and delve into the underlying profound research paradigm shifts. Ultimately, this work aims to provide scholars in the field with a macroscopic knowledge framework and offer deep insights into future trends.

## 2 Materials and methods

### 2.1 Data sources and search strategy

The Web of Science (WoS) Core Collection (WOSCC), developed by Clarivate Analytics, was selected as the primary data source due to its role as a globally authoritative citation database for natural sciences and clinical research. To ensure a comprehensive mapping of the research landscape, two researchers (YHZ and RFL) independently executed the literature search on September 5, 2025.

**Figure 1:**
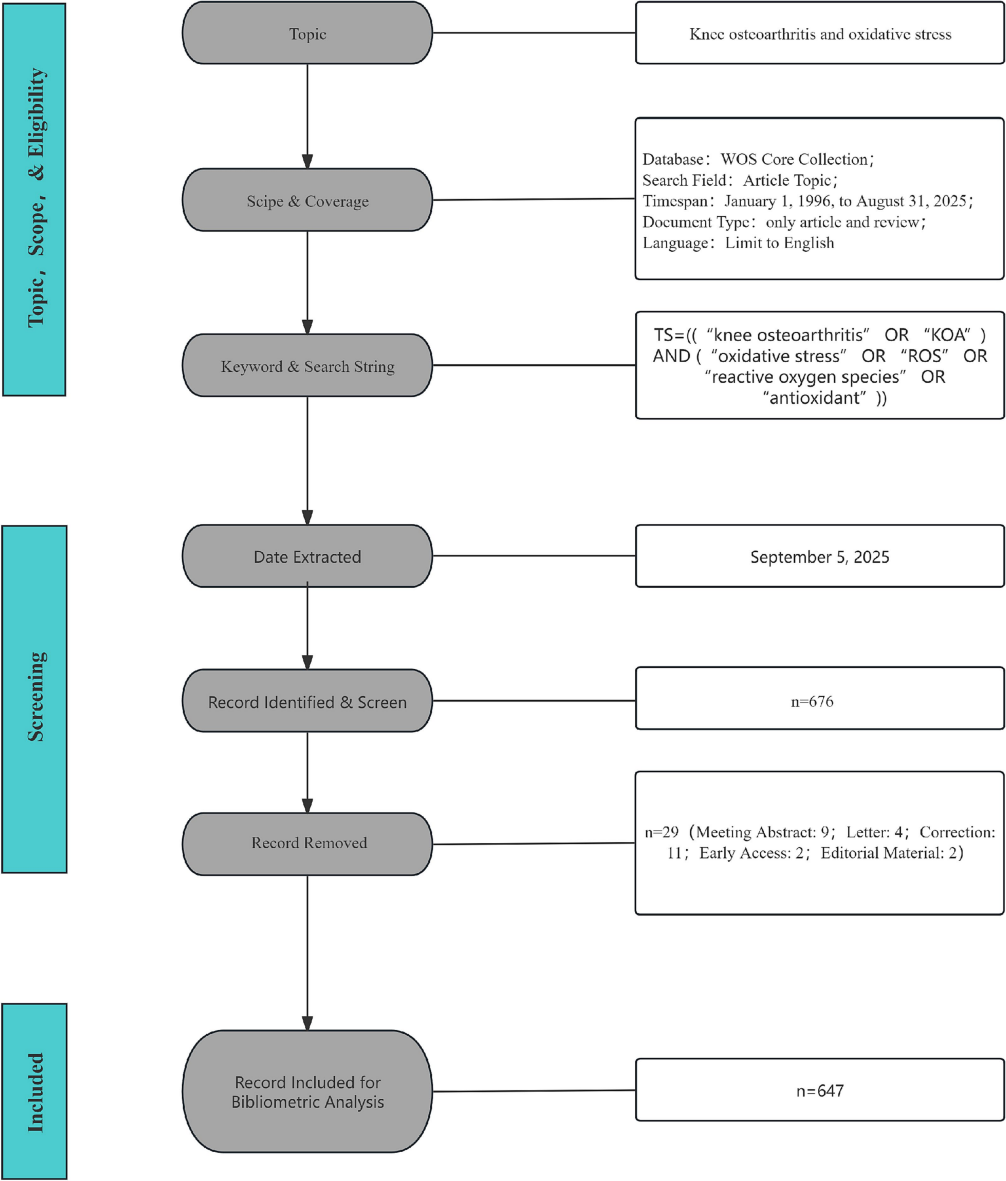
Flowchart of the literature selection process. The data were retrieved from the Web of Science Core Collection (WoSCC) on September 5, 2025, covering the period from 1996 to August 31, 2025. The chart illustrates the systematic screening process, including inclusion/exclusion criteria and the final document count (*N* = 647).

The search query was constructed as follows: TS = ((“knee osteoarthritis” OR “KOA”) AND (“oxidative stress” OR “ROS” OR “reactive oxygen species” OR “antioxidant”)). The time span was set from January 1, 1996, to August 31, 2025. Regarding the research subjects, we implemented an inclusive strategy to capture the complete evolutionary trajectory of the field. This study included human clinical trials, animal model studies, and both *in vitro* (cellular) and *in vivo* experimental research. By incorporating these diverse study types, we aimed to map the transition from foundational mechanistic validation to clinical translational expansion. As shown in Figure 1, the document types were restricted to “Articles” and “Reviews.” The language was strictly limited to publications where the full text was available in English; records that provided only an English abstract while the body text was in another language were excluded to ensure the precision of the subsequent bibliometric parsing and content analysis. The preliminary search retrieved 676 relevant records.

### 2.2 Literature screening and data processing

To minimize selection bias, a standardized two-stage screening process was implemented. The screening was performed independently by two researchers (YHZ and RFL) rather than under supervision, ensuring that each record was evaluated from two professional perspectives.

In the first stage, titles and abstracts were screened to exclude irrelevant studies, duplicate records, conference abstracts, letters, and editorial materials. In the second stage, the full texts of the remaining articles were retrieved and scrutinized based on the predefined inclusion and exclusion criteria. Any inconsistencies or discrepancies between the two investigators regarding the eligibility of a study were resolved through consensus-based discussion. If a consensus could not be reached, a third senior researcher was consulted to make the final determination. To assess the consistency of the screening results, Cohen’s kappa coefficient (*κ*) was calculated, yielding a value of 0.85, which indicates a high level of inter-rater reliability.

During data processing, all references were imported into Endnote X9.3.3 for manual record-by-record verification of metadata, including titles, authors, DOI, and journal information. Following the exclusion of 29 records (as detailed in Figure 1, including meeting abstracts, letters, and corrections), a total of 647 unique, high-quality full-text English articles and reviews were finalized for visual analysis using CiteSpace, VOSviewer, and RStudio.

### 2.3 Data analysis tools and methods

To provide a comprehensive and multi-dimensional bibliometric perspective, this study employed a synergistic approach using CiteSpace, VOSviewer, and RStudio. Rather than redundant mapping, each tool was selected for its unique analytical strengths to address specific research questions.

CiteSpace was primarily utilized to capture the dynamic evolution and emerging frontiers of the field (13), a task for which its time-slicing and burst-detection algorithms are specifically optimized. For these analyses, the parameters were configured with a time span from 1996 to 2024 divided into 1-year slices, where keywords served as the node type and the Top 25 most frequent entries per slice were selected. To optimize the network structure and enhance clarity, the Pathfinder and Pruning sliced networks algorithms were applied as the primary pruning methods. The reliability and effectiveness of the resulting clustering were validated by Modularity and Weighted Mean Silhouette values that surpassed established benchmarks, thereby ensuring both the structural significance and the internal consistency of the visual findings.

While CiteSpace focuses on temporal dynamics, VOSviewer was employed for its superior capability in structural network layout and density visualization, providing a clear overview of the collaborative landscape (14). VOSviewer was used to construct Co-authorship Networks (at author, institution, and country levels) and Co-citation Networks. Unlike CiteSpace’s keyword clustering, VOSviewer’s Keyword Co-occurrence Analysis was specifically used to map the global “knowledge distance” between core concepts, utilizing a frequency threshold (*n* ≥ 50) to ensure network clarity and focus on high-impact themes.

RStudio (specifically the bibliometrix and ggplot2 packages) served as the statistical engine to supplement the visual networks with rigorous quantitative data and customized high-quality graphics, such as bar charts, word clouds, dendrograms, rose diagrams, and geographical maps (15). RStudio was used to perform precise descriptive bibliometric analysis, such as calculating annual growth rates, identifying the most productive countries via geographical mapping, and generating Three-Field Plots to synthesize the relationships between authors, keywords, and journals. This provided a statistical foundation that the network-centric tools (CiteSpace/VOSviewer) cannot offer in isolation.

By integrating these three tools, this study ensures a holistic analysis: VOSviewer defines the structural breadth of the field, CiteSpace identifies the temporal depth and future directions, and RStudio provides the quantitative precision required for a robust bibliometric review.

## 3 Results

### 3.1 Annual publication trend analysis

**Figure 2:**
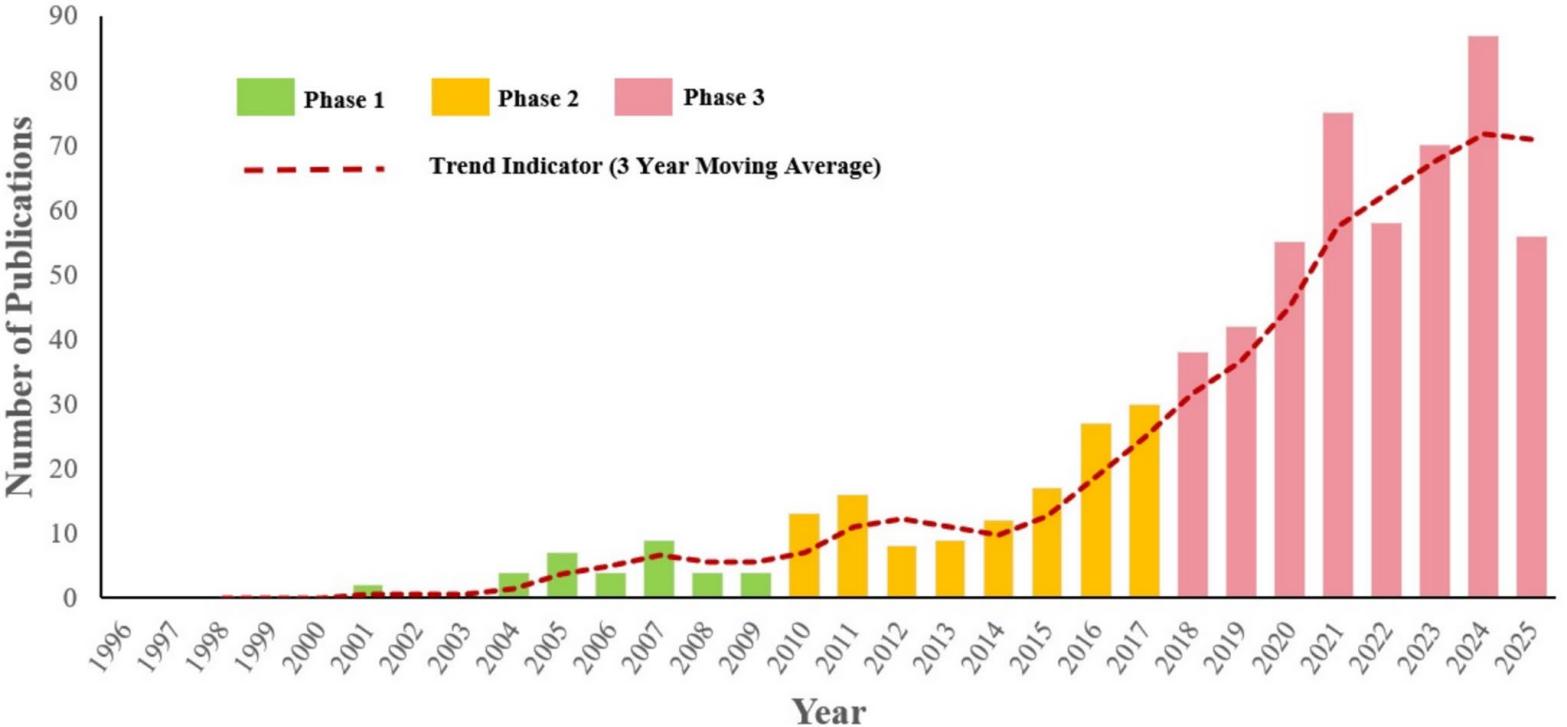
Annual distribution and publication trends. Trend chart of publication year and volume of academic publications in the field of KOA and oxidative stress research.

The temporal distribution of annual publication volume, visualized as a bar chart (Figure 2), clearly illustrates the overall developmental trajectory of the field. Research activity in KOA and oxidative stress from 1996 to 2025 demonstrated distinct phase-based evolutionary characteristics. The first phase (1996–2009) represented a nascent stage, with an annual average of merely 3.4 publications (standard deviation = 2.2). The second phase (2010–2015) marked a period of stable growth, during which the annual average increased to 12.2 publications (standard deviation = 4.5). The third phase (2016–2025) was characterized by rapid expansion, with the annual average rising significantly to 52.0 publications (standard deviation = 20.1). After applying a three-year moving average for smoothing, a consistent upward trend was observed from 2016 to 2019 (average annual increase of 6.5 publications), followed by a phase of accelerated growth from 2020 to 2022 (average annual increase of 12.3 publications). Notably, the year 2024 saw the highest number of publications (*n* = 87) in the recorded period. Furthermore, the higher standard deviation in the third phase suggests the field is evolving towards greater diversification and depth. This trend reflects the growing interest of the scientific community in the mechanisms of KOA and oxidative stress and indicates the field’s progressive maturation.

### 3.2 Country/region contribution analysis

**Figure 3:**
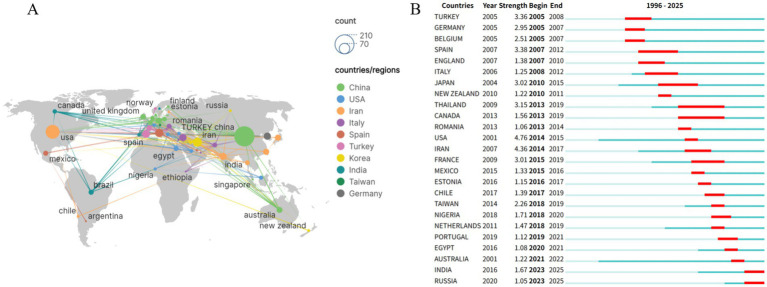
Global distribution and citation impact by country/region. **(A)** Geographical distribution map of national/regional contribution: the size of the circle reflects the number of documents issued by the country or region. The larger the circle, the more documents issued; the thickness and density of the line reflect the network diagram of the depth and frequency of cooperation between countries or regions. The thicker the line, the closer the cooperation. **(B)** Top 25 Countries/Regions with strongest citation bursts: the dark blue line traces each country’s or region’s research timeline, with the red portion highlighting the duration and impact of citation bursts. Light blue indicates periods without publications in this field.

Using RStudio, we generated a geographical distribution map (Figure 3A) of the relevant literature in the KOA oxidative stress research field based on the corresponding author’s country data. The size and color of the circles differentiate the contribution levels of individual countries. Globally, 66 countries/regions have participated in KOA oxidative stress research. China, the USA, Iran, and Italy were the top four contributing countries, publishing 209 (24.82%), 100 (11.88%), 42 (4.99%), and 36 (4.28%) publications, respectively. Figure 3B highlights the top 25 countries/regions with the strongest citation bursts in this field over the past three decades, with India and Russia emerging as research hotspots in recent years. Regarding centrality (Table 1), the USA (centrality 0.18), Iran (centrality 0.31), and Italy (centrality 0.62) play pivotal hub roles in the collaboration network, indicating strong international influence. Although China leads in publication volume, its relatively low centrality (0.06) suggests potential for further enhancing international collaboration. Spain, Turkey, Korea, and India are also prominent contributors, collectively forming a tripartite research landscape centered on the Asia-Pacific, North America, and Europe.

**Table 1:** The top 10 high-producitivity countries/regions.

Rank	Countries/regions	Count	Centrality	Year
1	China	209	0.06	2009
2	USA	100	0.18	2001
3	Iran	42	0.31	2007
4	Italy	36	0.62	2006
5	Spain	30	0.11	2007
6	Turkey	27	0	2005
7	Korea	27	0	2013
8	India	26	0.2	2016
9	Taiwan	20	0.08	2014
10	Germany	20	0.37	2005

### 3.3 Institutional collaboration network analysis

**Figure 4:**
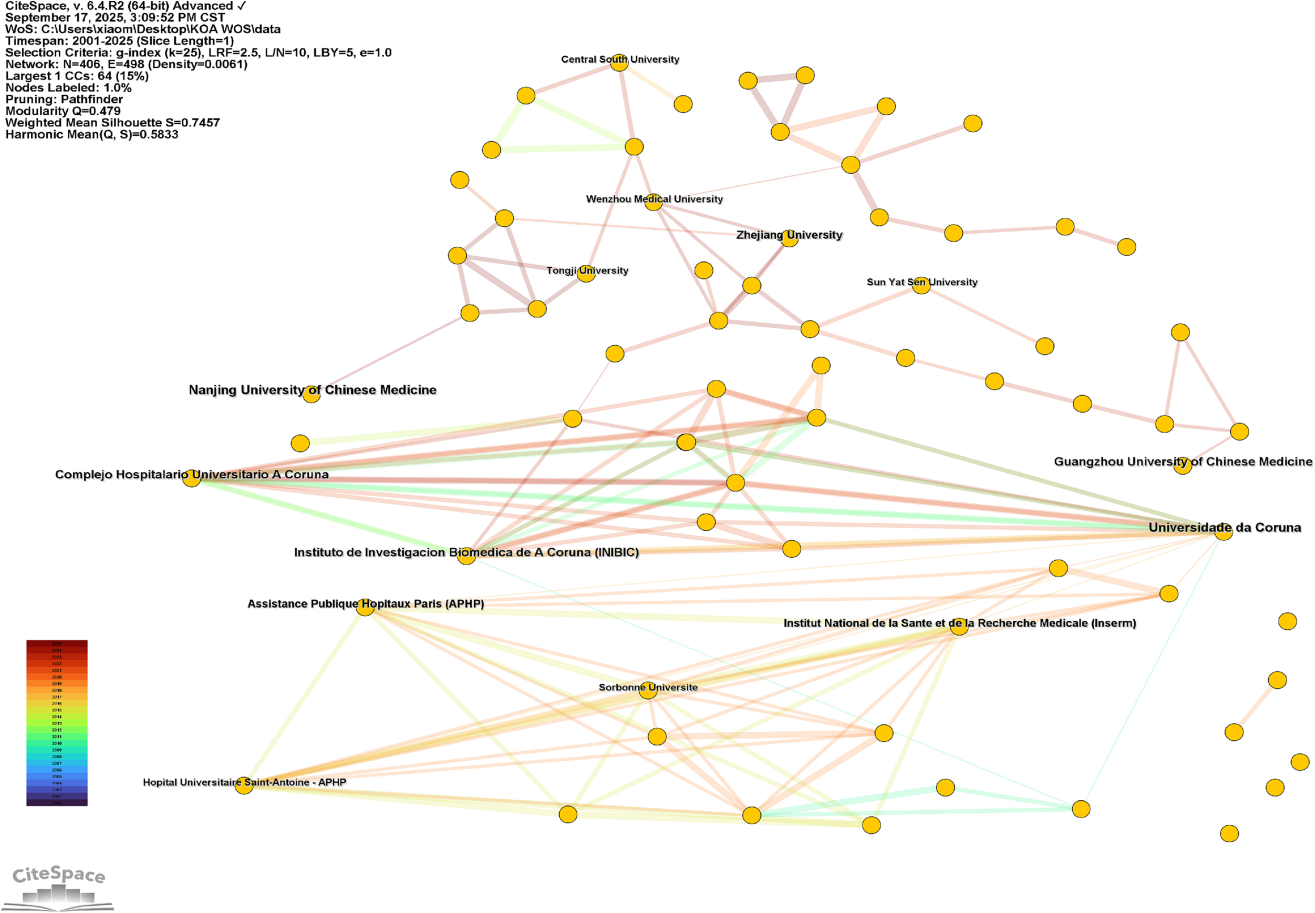
Institutional collaboration network generated by CiteSpace. Institutional cooperation network diagram. The nodes in the figure represent the research institutions, and the connection between the nodes represents the cooperation relationship between the institutions.

To elucidate collaborative relationships and identify the distribution of core organizations, we conducted a combined visual analysis using CiteSpace and VOSviewer. The CiteSpace network (Figure 4) comprises 406 nodes and 498 links, with a remarkably low network density of 0.0061. To contextualize this value, bibliometric studies on similar musculoskeletal or degenerative disease fields report low institutional collaboration network densities in CiteSpace analyses, reflecting generally sparse inter-institutional cooperation. For example, a bibliometric study of osteoporosis and nutrition research (16) using CiteSpace reported an institutional collaboration network density of 0.0105 among author networks, indicating relatively weak connectivity among institutions in that field. Our finding of 0.0061 sits at the lower end of this benchmark spectrum, suggesting that while the field of oxidative stress in KOA has reached a certain scale of maturity, it remains structurally fragmented compared to more integrated clinical fields.

This sparse connectivity indicates that research is currently characterized by a “transitional maturation” phase. In this phase, a vast number of institutions are active, but the field lacks a unified global consortium, resulting in most organizations operating in relative isolation. The low density reflects a “disciplinary lag,” where researchers in foundational molecular biology and those in clinical orthopedics may be working in parallel without sufficient cross-institutional integration. Furthermore, while the modularity *Q*-value of 0.479 confirms a pronounced community structure—indicating that cooperation is concentrated within specific, highly-specialized research “silos”—the lack of inter-silo connections keeps the overall density depressed.

**Figure 5:**
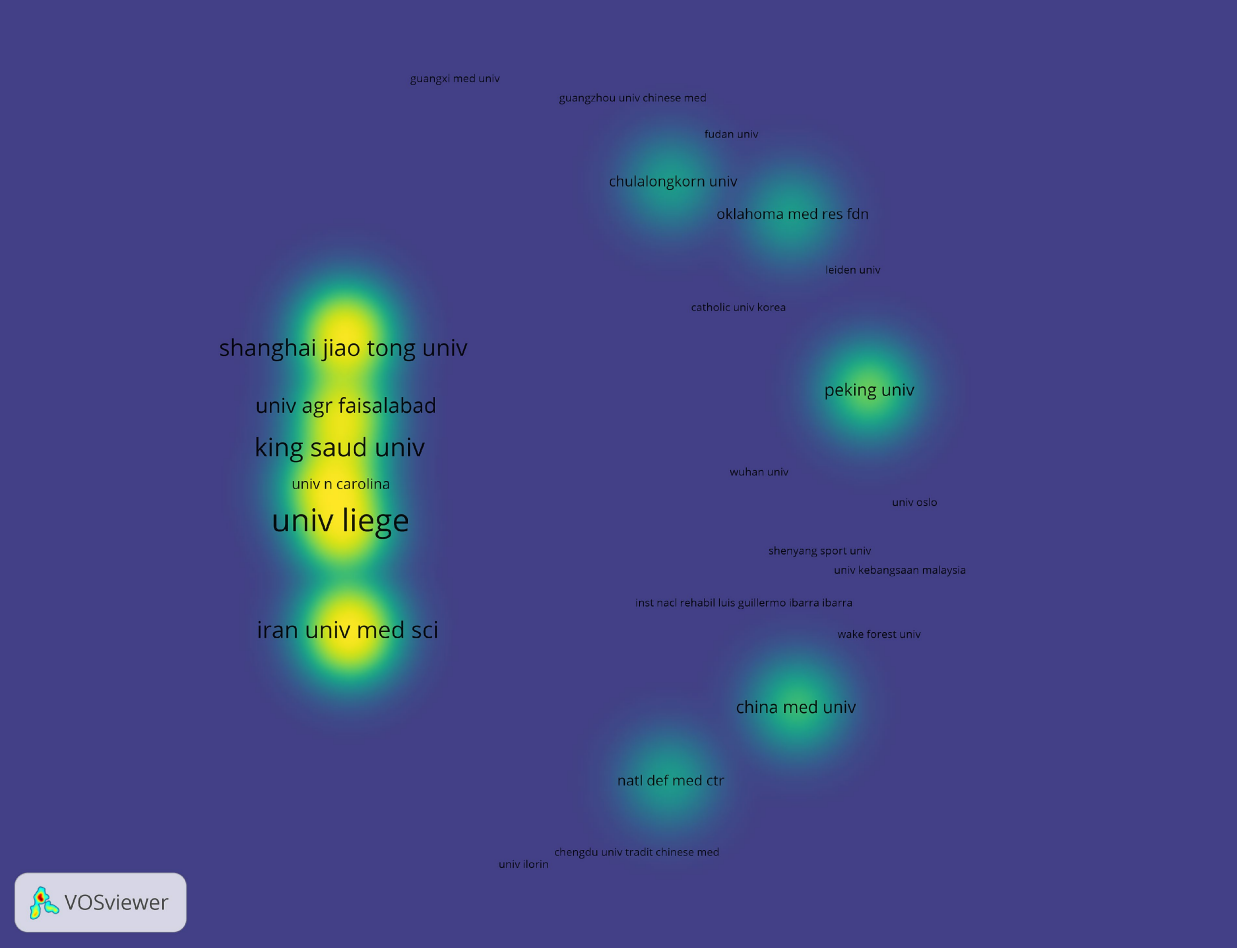
Institutional density visualization map by VOSviewer. Density map of institutions/organizations. The more yellow and green the color, the higher the activity and contribution of the representative institutions in the field of KOA oxidative stress research.

A primary driver of this low overall density is the “high output, low centrality” paradox observed in major contributing regions. As shown in the VOSviewer heat map (Figure 5) and Table 2, Chinese institutions like Shanghai Jiao Tong University and Nanjing University of Chinese Medicine exhibit the highest publication volumes but maintain disproportionately low betweenness centrality. This suggests that the massive volume of research produced in these regions is largely driven by intensive internal (domestic) collaboration or large-scale independent productivity within a “self-contained” research ecosystem. Because these high-output clusters do not frequently bridge to international partners, they fail to generate the “links” necessary to increase the global network density.

**Table 2:** Number of publications top 10 institutions.

Rank	Institution	Freq	Centrality	Country
1	Universidade da Coruna	13	0.05	Spain
2	Nanjing University of Chinese Medicine	13	0.10	China
3	Egyptian Knowledge Bank (EKB)	12	0.03	Egypt
4	Instituto de Investigacion Biomedica de A Coruna (INIBIC)	11	0.02	Spain
5	Complejo Hospitalario Universitario A Coruna	10	0.09	Spain
6	Guangzhou University of Chinese Medicine	10	0.10	China
7	Tabriz University of Medical Science	9	0.03	Egypt
8	Assistance Publique Hopitaux Paris (APHP)	8	0.01	France
9	Institut National de la Sante et de la Recherche Medicale (Inserm)	8	0.02	France
10	Zhejiang University	8	0.03	China

In contrast, Spanish institutions such as Universidade da Coruña and Instituto de Investigación Biomédica de A Coruña (INIBIC) occupy critical hub positions with high centrality despite lower total output. These organizations serve as vital bridges, but their influence is not yet sufficient to consolidate the fragmented global network. This structural landscape suggests that the field’s future development depends less on increasing publication volume and more on breaking down these regional and disciplinary silos. To enhance global knowledge exchange, high-output regions like China must transition from internal intensive growth to deeper structural integration within the international collaborative core.

### 3.4 Author co-occurrence network analysis

**Figure 6:**
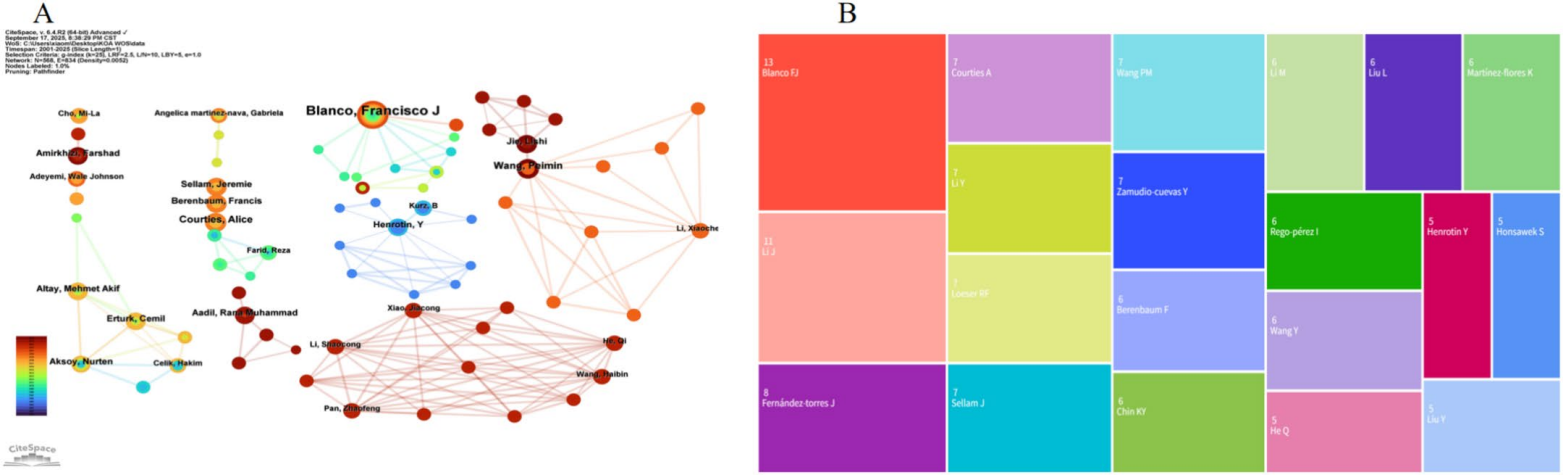
Author collaboration and contribution profiles. **(A)** Author co-occurrence network diagram. In the figure, the node represents the research author, the color represents the publication time, and the connection between the nodes represents the cooperation relationship between the authors. **(B)** The author contributed the first 20 trees. The larger the rectangular area, the greater the proportion of the author’s contribution.

The author collaboration network (Figure 6A) comprises 686 nodes and 9,434 edges, with a network density of 0.0052. This indicates a large number of participating authors in the field, while suggesting potential for further improvement in overall collaborative connectivity. The network exhibits a multi-cluster structure, featuring several distinct collaborative subgroups. These include a pivotal hub-centered cluster around Blanco, Francisco J.; densely interconnected teams represented by authors such as Jie Lishi and Wang Peimin; and smaller research groups formed around investigators like Henrotin, Y. and Kurz, B. This structure reflects the collaborative characteristics of clustering and diversity within the field.

The dendrogram of the top 20 contributing authors (Figure 6B) reveals a clear hierarchical distribution among core researchers. Blanco FJ occupies the leading position, indicated by the largest node area. Authors including Li J and Fernández-Torres J constitute a significant contribution tier, while other researchers such as Courties A, Wang PM, and Henrotin Y represent varying levels of output, collectively supporting the development of the field. Furthermore, the core author group encompasses researchers from multiple countries, demonstrating a marked international collaborative feature. In summary, author collaboration in KOA oxidative stress research is characterized by coexisting multiple clusters guided by hub authors, with the core researcher community exhibiting both hierarchy and international diversity, providing crucial support for the advancement of the field.

### 3.5 Keyword analysis

**Figure 7:**
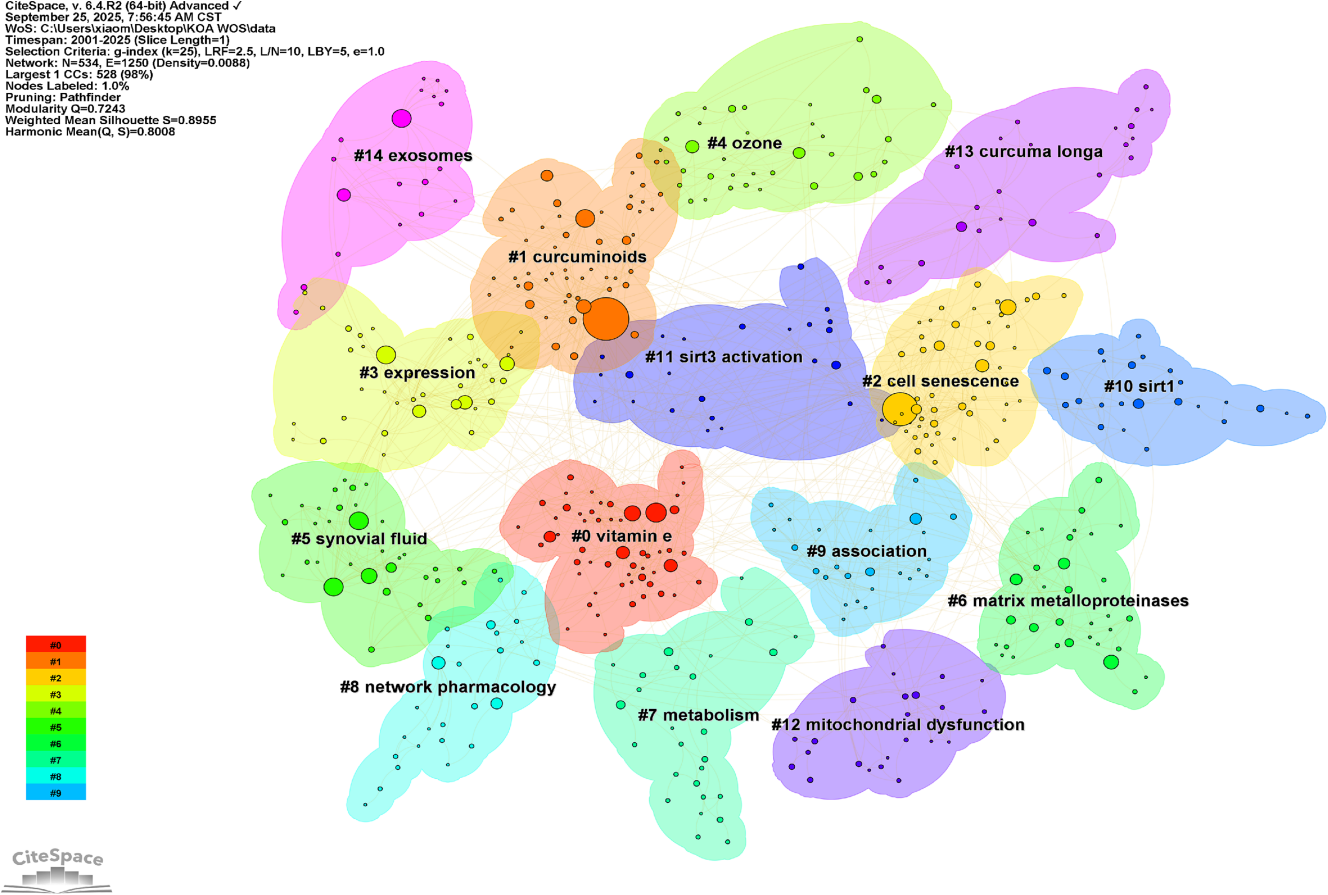
Thematic clustering of keywords based on CiteSpace. Top 15 cluster diagram of keywords. Different colors represent different clustering groups, and each clustering group is labeled with its representative term.

Keywords represent the core content of research literature and can effectively condense central research themes. Cluster analysis groups keywords into thematic categories based on similarity aiding researchers in intuitively understanding the knowledge structure hot topics and development trends of a research field (17). The keyword clustering for KOA and oxidative stress research (Figure 7) identified 15 primary clusters (*Q* = 0.7243 > 0.3, *S* = 0.8955 > 0.7) which reflect a broad research landscape spanning from long-standing nutritional interventions to maturing cellular mechanisms and clinical therapeutic approaches

**Figure 8:**
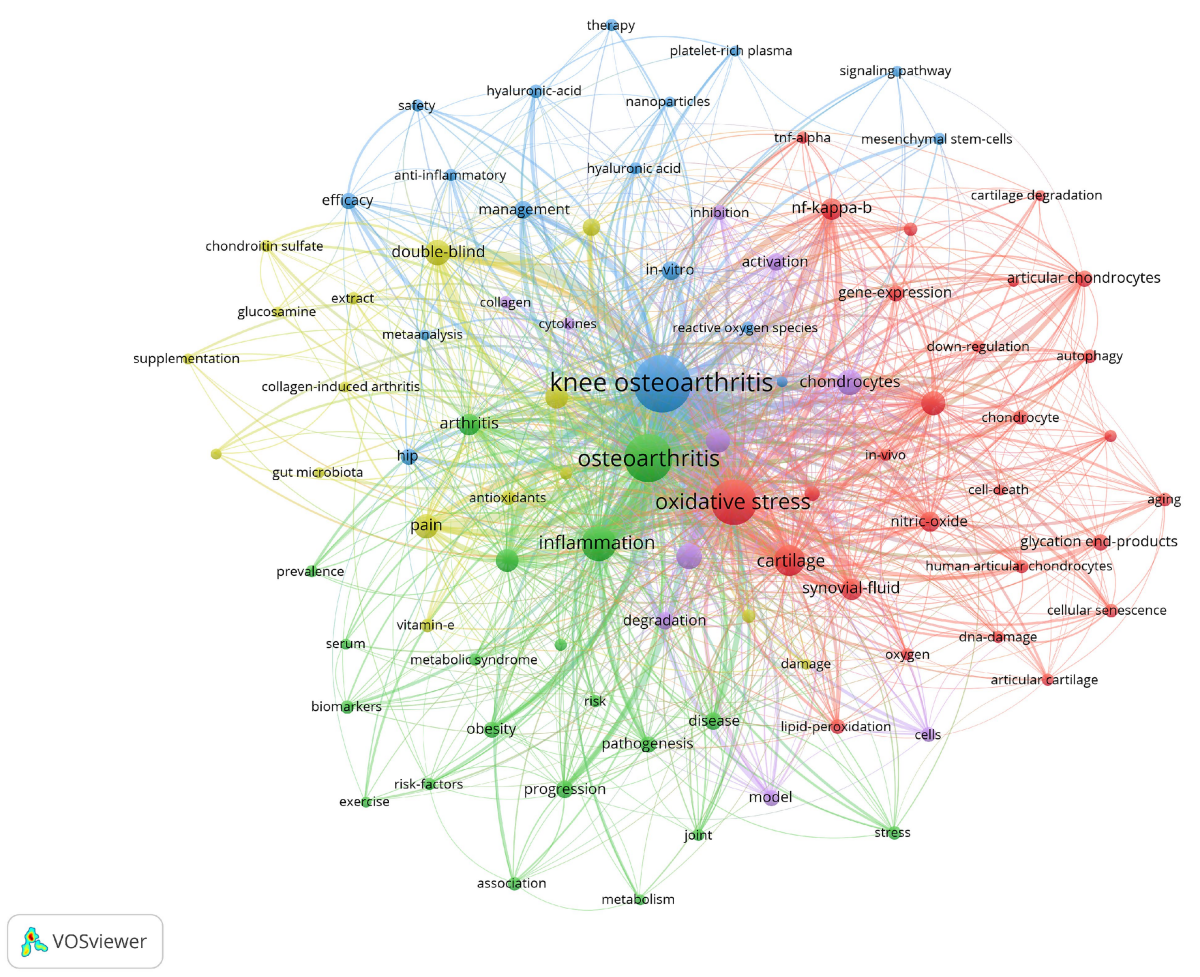
Keyword co-occurrence network by VOSviewer. Keyword co-occurrence diagram. The circle: representing the keyword, the circle size is proportional to the frequency of occurrence. Color: Correlation between keywords. Connection: The thickness of the connection represents the co-occurrence intensity of the keywords.

The largest cluster, #0 “vitamin e” (Size = 54, *S* = 0.887, Table 3), represents one of the most enduring and traditional research directions, focusing on the interventional role of vitamin E in KOA and oxidative stress research. Its representative keywords—vitamin e (10.98, 0.001), chondroitin sulphate (9.08, 0.005), and oxidative status (9.08, 0.005) (Table 3). The prominence of “vitamin e,” “chondroitin sulphate,” and “oxidative status” in Figure 8 confirms the sustained academic interest in nutritional supplements as a foundational strategy for cartilage protection. Biologically, Vitamin E acts as a critical lipid-soluble antioxidant that protects chondrocyte membrane integrity by neutralizing reactive oxygen species (ROS) and inhibiting lipid peroxidation (18). The clustering of “oxidative status” with “chondroitin sulphate” suggests that nutritional interventions are no longer viewed merely as supplements but as a consolidated strategy to modulate the intra-articular microenvironment, aiming to shield the extracellular matrix (ECM) from oxidative degradation (19).

**Table 3:** Top 5 keyword clustering in research field.

Cluster lD	Cluster name	Amount	Silhouette	The top three representative keywords in the LLR cluster
0#	Vitamin e	54	0.887	Vitamin e (10.98, 0.001); chondroitin sulphate (9.08, 0.005); oxidative status (9.08, 0.005)
1#	Curcuminoids	54	0.923	Curcuminoids (13.84, 0.001); systematic review (13.84, 0.001); double blind (12.97, 0.001)
2#	Cell senescence	53	0.874	Cell senescence (14.84, 0.001); DNA damage (11.12, 0.001); oxidative stress (10.02, 0.005)
3#	Expression	38	0.889	Expression (12.13, 0.001); biochemical markers (9.74, 0.005); selenium (9.74, 0.005)
4#	Ozone	36	0.868	Ozone (10.32, 0.005); systematic reviews (8.5, 0.005); platelet-rich plasma (8.5, 0.005)

LLR is the maximum likelihood algorithm, and the values in brackets are LLR value and *p*-value, respectively. The greater the LLR value, the stronger the correlation between keywords and clustering; the smaller the p value, the more significant the correlation between keywords and clustering.

Similarly, Cluster #1 “curcuminoids” (Size = 54, *S* = 0.923, Table 3) highlights a maturing thematic area centered on natural products. The close association between “curcuminoids (13.84, 0.001)” “systematic review (13.84, 0.001)” and “double blind (12.97, 0.001)” indicates that research in this sub-field has moved past the exploratory phase and is now firmly in the stage of evidence-based clinical validation. Mechanistically, curcuminoids are recognized for their dual role: scavenging ROS and activating the Nrf2/HO-1 signaling pathway, which upregulates endogenous antioxidant defenses. The clinical implication is profound, indicating that the research community is actively validating natural antioxidants as rigorous pharmacological candidates to mitigate the inflammatory-oxidative cycle in KOA patients (20).

In terms of mechanistic research, Cluster #2 “cell senescence” (Size = 53, *S* = 0.874, Table 3) underscores well-established pathological frameworks. The interconnectedness of cell senescence (14.84, *p* < 0.001), dna damage (11.12, *p* < 0.001), and oxidative stress (10.02, *p* < 0.005) in Figure 7 provides a bibliometric mapping of the “Oxidative Stress → Genomic Instability → Senescence-Associated Secretory Phenotype (SASP)” axis. This cluster reveals that oxidative stress is not merely a byproduct of KOA but a primary driver of chondrocyte “geroncogenesis” (21)—where accumulated DNA damage triggers a permanent cell-cycle arrest, leading to the secretion of pro-inflammatory cytokines and proteases that accelerate joint degeneration.

Methodological clusters such as Cluster #3 “expression” (Size = 38, *S* = 0.889) and Cluster #4 “ozone” (Size = 36, *S* = 0.868) further illustrate the integration of clinical and laboratory techniques. The presence of “biochemical markers (9.74, 0.005)” and “selenium (9.74, 0.005)” reflects the field’s ongoing reliance on established molecular detection methods. Cluster #4’s focus on “ozone (10.32, 0.005)” and “platelet-rich plasma (8.5, 0.005)” alongside “systematic reviews (8.5, 0.005)” suggests that these physical and biological therapies are also undergoing a phase of evidence synthesis, following years of clinical application.

**Figure 9:**
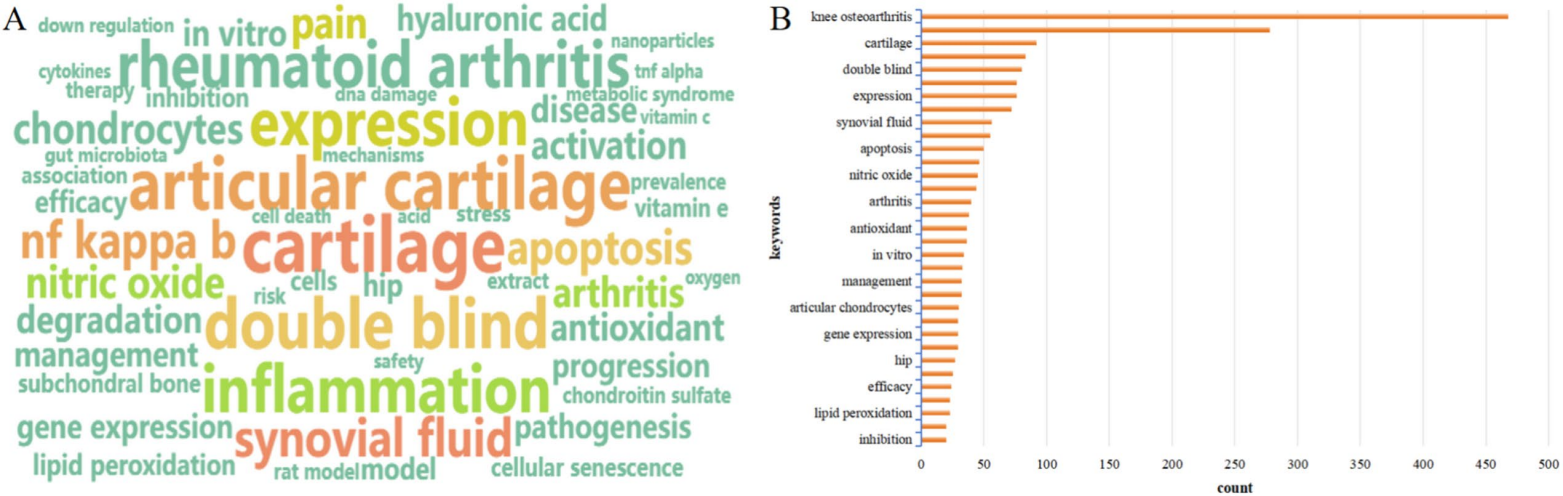
Quantitative synthesis of high-frequency keywords. **(A)** Keyword word cloud; font size reflects the frequency of occurrence in the 647 analyzed documents. **(B)** Frequency histogram of keywords appearing 20 times or more, identifying the dominant focal points in the field.

In summary, the keyword cloud (Figure 9A) and frequency histogram (Figure 9B, frequency≥20) reveal a multidimensional research landscape. This evolution demonstrates a clear trajectory: from early descriptive studies of “oxidative status” to the mechanistic dissection of “cell senescence,” and finally to the evidence-based validation of “antioxidant interventions.” This hierarchical structure confirms that oxidative stress remains the central link bridging basic chondrocyte biology to systemic clinical management in KOA.

### 3.6 Keyword timezone map and burst analysis

**Figure 10:**
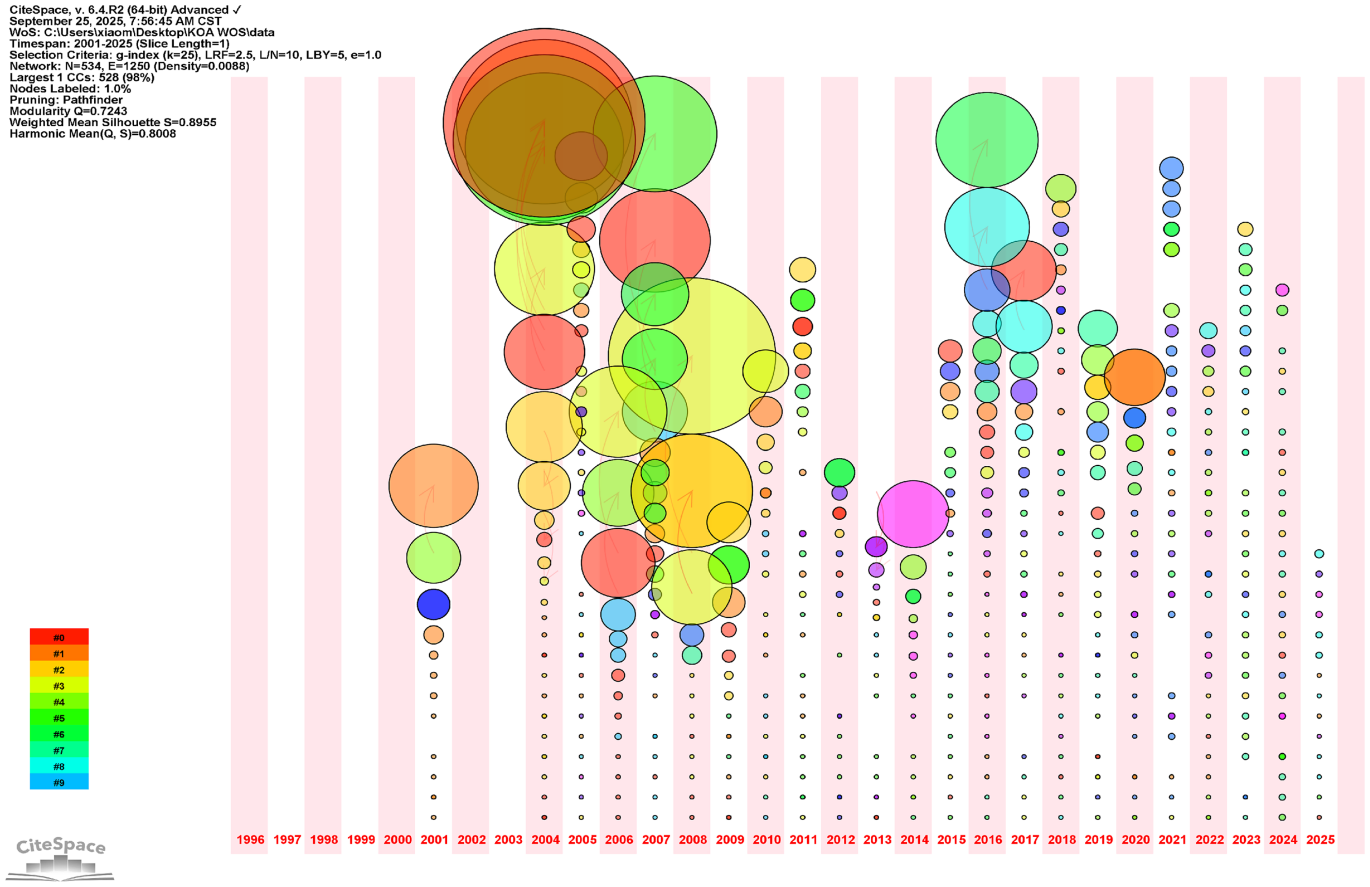
Chronological evolution of keywords (Timezone View). Keywords are arranged according to the year of their first appearance. This timezone map reveals the paradigm shift from foundational cartilage damage validation to systemic-local regulation and advanced delivery systems.

The keyword timezone map displays newly emerged keywords across different time periods, providing a temporal perspective on knowledge evolution and effectively identifying research hotspots and development trends in the field. Figure 10 visually presents the evolutionary trajectory of keywords in KOA and oxidative stress research along a chronological axis. Supported by network metrics (*Q* = 0.7243 > 0.3, indicating statistically significant cluster structure; mean silhouette *S* = 0.8955 > 0.7, indicating good intra-cluster homogeneity), distinct research phases can be clearly delineated: From before 1996 to approximately 2005 was an initial exploratory phase, characterized by a limited number of nodes focusing on validating the fundamental association between KOA and oxidative stress, laying the foundation for the field. From 2005 to 2015 marked a diversification and expansion phase, where increased node and cluster diversity reflected research branching into oxidative stress-mediated cellular/molecular mechanisms, biomarker identification, and nutritional/pharmaceutical interventions, demonstrating dual advancement in mechanistic understanding and intervention exploration. Post-2015 represents an innovation and deepening phase, maintaining research interest in classical themes like cartilage damage and core oxidative stress mediators while incorporating cutting-edge technologies such as omics and nanocarriers, reflecting a developmental pattern where classical themes persist alongside cutting-edge innovations.

**Figure 11:**
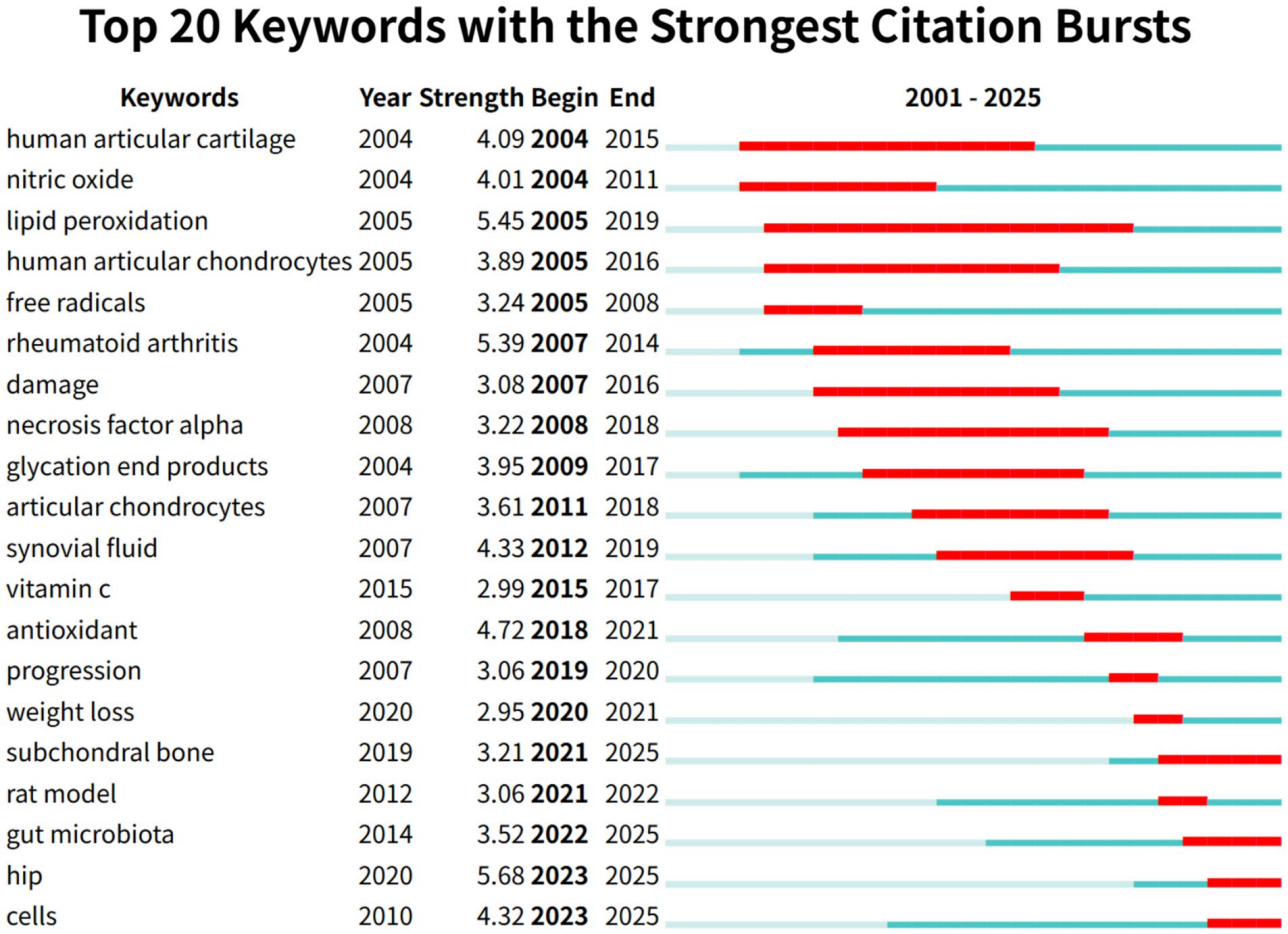
Top 20 keywords with the strongest citation bursts. The blue lines represent the 30-year timeline, while red segments mark the specific periods of rapid frequency increases. This identifies “lipid peroxidation” as the most sustained hotspot and “subchondral bone” and “gut microbiota” as emerging frontiers.

Keyword bursts refer to a sudden increase in the frequency of specific keywords within a certain period representing shifts in research focus and serving as indicators of research frontiers. Figure 11 reveals these explosive trends in research hotspots complementing the timezone map. In the early phase (2004–2010) bursts of keywords like human articular cartilage and nitric oxide anchored classical oxidative stress mediators and the target tissue (articular cartilage) validating the fundamental “oxidative stress → cartilage damage” association. During the middle phase (2011–2018) bursts of lipid peroxidation synovial fluid and antioxidant reflected deeper investigation into the cellular microenvironment (synovial fluid) and pathological processes (lipid peroxidation) alongside exploration of antioxidant intervention strategies. In the recent phase (2019–2025) bursts of subchondral bone gut microbiota and cells indicate the emergence of new targets (subchondral bone) interdisciplinary perspectives and advanced technologies. Notably lipid peroxidation exhibited the longest burst duration (2005–2019) underscoring its sustained research attention as a core pathological process in oxidative stress. Bursts in the last 3 years such as “hip” (reflecting oxidative stress research in hip osteoarthritis) and “cells” (pertaining to interventions using stem cells/exosomes and other cellular carriers) further highlight the field’s expansion towards interdisciplinary integration and precise intervention strategies.

## 4 Discussion

### 4.1 Collaborative characteristics and resource integration challenges in the research landscape

Visualizations of institutional collaboration and author co-occurrence networks reveal that the KOA oxidative stress field has established a fundamental structure characterized by international collaboration guided by core hubs. Research institutions (e.g., Nanjing University of Chinese Medicine, Universidade da Coruña) and leading authors (e.g., Blanco FJ, Li J) have forged research connections at the organizational and individual levels, respectively. Furthermore, the three-dimensional thematic structure emerging from keyword clustering—“Intervention Strategies - Pathological Mechanisms - Research Methods”—further corroborates the collaborative nature of research content within this framework.

However, the low density of institutional collaboration (0.0061) and limited cross-subgroup collaboration among authors highlight a significant shortcoming in resource integration. Specifically, different research teams have formed localized closed loops around specialized topics, leading to insufficient cross-disciplinary technological integration. This is particularly evident in the “high output, low centrality” phenomenon observed in Chinese institutions (Section 3.3). Despite high publication volumes, the lack of structural centrality indicates that these research outputs are not yet fully integrated into the global knowledge-sharing core. Future efforts should focus on establishing multinational alliances to dismantle these regional and disciplinary barriers.

### 4.2 Evolutionary logic and bibliometric-to-mechanistic mapping

The chronological evolution of keywords (Figure 10) and their corresponding burst dynamics (Figure 11) do not merely reflect shifting academic interests; they map a profound paradigm shift in the biological understanding of knee osteoarthritis (KOA). Supported by robust network metrics (*Q* = 0.7243, *S* = 0.8955), this evolution follows a clear, logical trajectory: Foundational Validation (Pre-2005) → Mechanistic Refinement (2005–2015) → Translational & Systemic Expansion (Post-2015). By anchoring these data-driven bibliometric clusters to established molecular pathways, we can decode the biological landscape of KOA and oxidative stress.

#### 4.2.1 Phase I (pre-2005): foundational validation and the “nitric oxide-cartilage” axis

In the nascent stage of this field, the primary scientific objective was to establish whether oxidative stress was a bystander or an active mediator in KOA pathogenesis. The early citation bursts of “human articular cartilage” and “nitric oxide” (Figure 11) serve as bibliometric anchors for this foundational validation.

During this period, researchers focused on identifying the primary reactive species within the joint cavity. Nitric oxide (NO), synthesized by inducible nitric oxide synthase (iNOS) in chondrocytes under inflammatory stimulation, emerged as a pivotal reactive nitrogen species (RNS) (22, 23). Mechanistically, excessive NO directly impairs chondrocyte viability by inhibiting the synthesis of type II collagen and proteoglycans, while simultaneously activating matrix metalloproteinases (MMPs) and inducing chondrocyte apoptosis (22, 24, 25). This early bibliometric node laid the groundwork for the field, proving that the intra-articular redox state directly dictates extracellular matrix (ECM) integrity and chondrocyte survival.

#### 4.2.2 Phase II (2005–2015): mechanistic refinement—from “lipid peroxidation” to “cell senescence”

As the field matured, the research focus transitioned from observing macro-level cartilage damage to dissecting micro-level cellular fates and endogenous defense loops. This phase is characterized by the longest-running burst of “lipid peroxidation” (2005–2019) and the consolidation of Cluster #2 (“cell senescence”) and Cluster #3 (“expression”) (Table 3).

##### 4.2.2.1 The “lipid peroxidation” to “cell senescence” transition

The sustained burst of “lipid peroxidation” highlights its role as a core pathological bridge. Reactive oxygen species (ROS) attack the polyunsaturated fatty acids of chondrocyte membranes, generating toxic lipid aldehydes such as malondialdehyde (MDA) and 4-hydroxynonenal (4-HNE). These byproducts not only compromise membrane permeability but also act as secondary messengers that trigger genomic instability.

This molecular cascade is mathematically encapsulated in Cluster #2 (*S* =0.874 ), which groups “cell senescence,” “dna damage,” and “oxidative stress.” Chronologically, this represents a paradigm shift: the field evolved from studying passive cellular “damage” (lipid peroxidation) to active cellular “fate” decisions (senescence). Accumulating DNA double-strand breaks induced by ROS activate the p53/p21 and p16 INK4a/Rb pathways (26), driving chondrocytes into a state of permanent cell-cycle arrest. These senescent chondrocytes acquire a Senescence-Associated Secretory Phenotype (SASP), actively secreting pro-inflammatory cytokines (IL-1 β , TNF- α ) and catabolic enzymes (MMP-1, MMP-13, ADAMTS-4, ADAMTS-5) (27, 28), which accelerate joint degeneration in a paracrine manner.

##### 4.2.2.2 The Nrf2/HO-1 antioxidant defense loop

This mechanistic refinement is further reflected in Cluster #3 (“expression” and “biochemical markers”). Under physiological conditions, the transcription factor Nrf2 is sequestered in the cytoplasm by Keap1 (29). Upon ROS exposure, Nrf2 should dissociate from Keap1, translocate to the nucleus, and bind to Antioxidant Response Elements (ARE) to upregulate endogenous antioxidant enzymes, including superoxide dismutase (SOD), catalase (CAT), and heme oxygenase-1 (HO-1) (30, 31).

However, in the chronic inflammatory microenvironment of KOA, this endogenous defense loop is severely compromised. The high co-occurrence of “biochemical markers” and “expression” in Cluster #3 reflects the intensive academic effort during this decade to quantify this failed antioxidant defense and to target the Nrf2/HO-1 axis as a therapeutic “master switch” to restore redox homeostasis.

#### 4.2.3 Phase III (post-2015): translational expansion and systemic-local interactions

Post-2015, the research landscape underwent a second major paradigm shift. Armed with a deep understanding of localized chondrocyte biology, researchers expanded their scope toward whole-joint pathology, systemic-local interactions, and advanced translational therapeutics. This is evidenced by the recent bursts of “subchondral bone,” “gut microbiota,” and “cells” (Figure 11), alongside the clinical evidence-synthesis clusters of Cluster #1 (“curcuminoids”) and Cluster #4 (“ozone”).

##### 4.2.3.1 The whole-joint paradigm (“subchondral bone”)

The recent burst of “subchondral bone” (2021–2025) signifies the transition from a “cartilage-centric” view to a “whole-joint” disease model. Under mechanical overload and chronic inflammation, oxidative stress in subchondral bone osteoblasts and osteoclasts drives abnormal bone remodeling (32). ROS accumulation promotes osteoclastogenesis via the RANKL/OPG pathway, leading to early-stage subchondral bone resorption and late-stage sclerosis (33). This aberrant remodeling releases angiogenic factors (such as VEGF) (34) and sensory nerve-stimulating factors (such as NGF) (35) through the subchondral plate into the cartilage, directly contributing to joint pain and accelerating overlying cartilage degradation.

##### 4.2.3.2 The gut-joint Axis (“gut microbiota”)

The burst of “gut microbiota” (2022–2025) represents the frontier of systemic-local regulation. Gut dysbiosis impairs intestinal barrier integrity, leading to the leakage of lipopolysaccharides (LPS) into the systemic circulation (36). Systemic low-grade endotoxemia primes synovial macrophages and chondrocytes, upregulating NADPH oxidases (NOXs) and mitochondrial ROS production. This “gut-joint axis” demonstrates that intra-articular oxidative stress is not merely a localized mechanical consequence but is actively modulated by systemic metabolic and immunological states (37).

##### 4.2.3.3 Advanced delivery and cellular therapies (“cells”)

The burst of “cells” (2022–2025) reflects the cutting-edge integration of nanotechnology and regenerative medicine. To overcome the rapid clearance and low bioavailability of traditional antioxidants in the joint cavity, researchers are utilizing mesenchymal stem cells (MSCs) and MSC-derived (38, 39) exosomes as natural nanocarriers. These exosomes deliver antioxidant microRNAs (e.g., miR-140, miR-21) (40) and proteins directly to damaged chondrocytes, effectively rescuing mitochondrial dysfunction, promoting mitophagy, and scavenging intracellular ROS.

##### 4.2.3.4 Evidence-based clinical translation

Finally, the clinical maturation of these antioxidant strategies is mathematically validated by Cluster #1 (“curcuminoids,” *S* = 0.923) and Cluster #4 (“ozone,” *S* = 0.868), both of which are dominated by the terms “systematic review” and “double blind.” This indicates that natural polyphenols (such as curcumin, which dual-targets Nrf2 activation and NF- κ B inhibition) (41) and biological therapies (such as ozone and platelet-rich plasma, which modulate the local redox microenvironment) (42, 43) have successfully transitioned from *in vitro* mechanistic validation to rigorous, evidence-based clinical evaluation.

### 4.3 Clinical translation gaps and future directions in research value

Despite significant progress in understanding the fundamental mechanisms of KOA and oxidative stress, the field continues to face considerable challenges in clinical translation. Keyword analysis reveals a notable disconnect between basic research and clinical application, characterized by the high frequency of terms like “systematic review” and “double blind” (Clusters #1, #4), in stark contrast to the conspicuous absence of translation-oriented themes such as “clinical diagnostic criteria for KOA oxidative stress.”

This translation gap manifests in two key dimensions. First, there is insufficient clinical applicability of biomarkers. Indicators identified under the “biochemical markers” cluster (Cluster #3), such as selenium and oxidative++ status, predominantly originate from laboratory studies. The lack of validation in large-scale clinical cohorts makes it difficult to establish them as reliable standards for diagnosis or efficacy evaluation. Second, translational efficiency of intervention strategies remains low. Although evidence-based support exists for interventions like curcuminoids (Cluster #1) and ozone therapy (Cluster #4), their integration into routine clinical practice is significantly hampered by the absence of systematic clinical optimization regarding “dosage-efficacy-safety” profiles.

To bridge this gap, we propose the following actionable strategies grounded in our findings:

1. Development of Standardized Biomarker Panels: Based on the high-impact keywords in Cluster #3 (biochemical markers) such as “selenium” and “oxidative status,” future research should prioritize large-scale clinical trials to establish these as validated diagnostic tools. The current absence of “diagnostic standards” in our keyword map suggests a clear direction for consensus-building studies.
2. Standardizing Natural Product Interventions: Research in Cluster #1 (curcuminoids) has reached the “double-blind” stage. However, the lack of keywords related to “pharmacokinetics” or “bioavailability” suggests that future bibliometric shifts should focus on optimizing “dosage-efficacy” profiles to move these therapies into routine clinical practice.
3. AI-Driven Precision Mapping: As identified in our discussion of “systemic-local” regulation, integrating AI (represented by the emerging “technology” trend) with multi-omics data can identify previously overlooked drivers of oxidative stress, accelerating the discovery of targeted therapies.

### 4.4 Limitations

While this study provides a comprehensive landscape of the KOA oxidative stress field by integrating CiteSpace, VOSviewer, and RStudio, several limitations must be acknowledged to ensure a nuanced interpretation of the findings:

1. Single Database Reliance and Selection Bias: We relied exclusively on WoSCC to ensure access to the citation metadata required for complex visualizations (e.g., burst detection). However, excluding databases like Scopus, PubMed, or Embase may omit relevant clinical or specialized studies, making this a representative rather than exhaustive landscape.
2. Linguistic and Geographic Bias: The focus on English-language publications may underrepresent regions like China, where significant research is published in local databases (e.g., CNKI). This potentially undervalues localized collaborative networks and regional therapeutic interventions.
3. Metrics vs. Quality: Bibliometric indicators measure academic “visibility” and “impact” (via citations and centrality) rather than the methodological rigor or intrinsic quality of a study. A identified “hotspot” signifies research intensity but does not inherently guarantee high-level clinical evidence.
4. Keyword and Temporal Bias: Inconsistencies in terminology (e.g., synonyms for mitochondrial pathways) may affect cluster precision. Furthermore “citation latency” means impactful papers from the last 1–2 years may not yet appear as hubs causing a slight delay in identifying the most recent frontiers
5. Subjectivity and Lack of Clinical Validation: Cluster interpretation remains partially subjective despite robust statistical metrics (S-value/Modularity Q). Crucially, bibliometric trends reflect research interest rather than proven clinical efficacy, necessitating future clinical trials to validate these findings for bedside application.

## 5 Conclusion

Over the three decades from 1996 to 2025, research on oxidative stress in knee osteoarthritis has undergone a remarkable scientific journey. Through the construction and interpretation of CiteSpace knowledge maps, we have witnessed the field’s evolution from an initial establishment of associations, through a phase of mechanistic deepening, and finally into a period focused on clinical translation. Correspondingly, the research paradigm has undergone a profound transformation—from descriptive and mechanistic investigations to systemic and therapeutically oriented approaches. Oxidative stress is no longer regarded as an isolated pathological component of KOA but is now recognized as part of a complex, dynamic molecular regulatory network, serving as a central hub linking aging, inflammation, and cartilage degeneration. Looking ahead, a new era of KOA treatment based on the precise regulation of oxidative stress is emerging, offering renewed hope for overcoming this persistent degenerative disease.

## Data Availability

The original contributions presented in the study are included in the article/supplementary material, further inquiries can be directed to the corresponding author.
